# Combined Therapy of Probiotic Microcapsules and Bomidin in *Vibrio parahaemolyticus*–Infected Rats

**DOI:** 10.3390/life12111740

**Published:** 2022-10-29

**Authors:** Ting Zhou, Mengting Liu, Jialiang Pan, Jianluan Ren, Fang Tang, Jianjun Dai, Feng Xue, Dejun Ji

**Affiliations:** 1 International Joint Research Laboratory of Animal Health and Food Safety, Key Laboratory of Animal Bacteriology, Ministry of Agriculture, College of Veterinary Medicine, Nanjing Agricultural University, Nanjing 210095, China; 2 Department of Aquaculture, Animal Science DEPT, Yangzhou University, Yangzhou 225100, China

**Keywords:** *Lacticaseibacillus casei* CGMCC1.8727, antimicrobial peptide, *Vibrio parahaemolyticus*, probiotic therapy, microcapsule

## Abstract

Background: With the discovery of more and more drug–resistant bacterial strains, there is an urgent need for safer and more effective alternative treatments. In this study, antibacterial peptides and probiotic microcapsules were combined to treat gastrointestinal inflammation caused by *Vibrio parahaemolyticus* infection. Methods: To improve the stability of probiotics in the gastrointestinal tract, two types of mixed natural anionic polysaccharides and chitosan were used as carriers to embed the probiotics. Taking *Lacticaseibacillus casei* CGMCC1.8727 microcapsules with good performance as the research object, the in vitro characteristics of the microcapsules were studied via acid resistance test and intestinal release test. The microcapsules were then tested for in vivo treatment in combination with the antibacterial peptide, bomidin, and the therapeutic effects were compared among microencapsulated probiotics, free probiotics, and probiotics in combination with bomidin. Results: Microencapsulation was successfully manufactured under suitable processing parameters, with the product particle size being 2.04 ± 0.2743 mm. Compared with free probiotics, microencapsulation significantly improved the activity and preservation stability of the probiotics under simulated gastrointestinal conditions. Microencapsulated probiotics showed better therapeutic effects than free probiotics in vivo. Microcapsules combined with antimicrobial peptides accelerated the elimination of bacteria in vivo. This study provides a reference for anti–inflammatory treatment, especially for the treatment of gastrointestinal diseases.

## 1. Introduction

*Vibrio parahaemolyticus* (also known as *Vibrio enteritidis*) infection causes acute gastroenteritis with abdominal pain, watery diarrhoea, nausea, vomiting and high fever, potentially leading to dehydration, shock, coma and even death [1]. Generally, antibiotics are the primary choice to treat bacterial diseases, while in recent years, the problem of antibiotic resistance has become increasingly serious due to excessive or unreasonable use of antibiotics. The diversification and development of antibiotics that do not easily produce drug resistance are urgently required [2].

Probiotics contain three groups: *bifidobacterium*, *lactobacillus* and *facultative anaerobic cocci*. There are 32 species in the *Bifidobacterium* genus, among which only 5 kinds of probiotics are allowed in human use. There are 56 species in the *Lactobacillus* genus, among which 3 kinds with good clinical records, and another 10 kinds that are mainly used as probiotics. The facultative anaerobic cocci genus includes more than five species [3], among which only a couple are used as probiotics, such as *Lacticaseibacillus casei* [4] *CGMCC1.8727* [5], which can inhibit the growth of pathogenic bacteria [6]*,* reduce blood pressure and cholesterol [7], promote cell division, enhance immunity, and prevent cancer [8]. It can also increase the number of lymphocytes, significantly enhance the macrophage function of lymphocytes, enhance the anti–virus ability of the body, accelerate the removal of bacteria in the intestinal tract and regulate intestinal microbiota to prevent acute diarrhoea and allergies. Because of its good probiotic effects on nutrition, immunity [9], and disease prevention, *Lacticaseibacillus casei* CGMCC1.8727 has become a focus of research, development, and production. With an increasing number of patients reporting enteritis–related diseases, it is of great significance to study *Lacticaseibacillus casei* CGMCC1.8727 and develop functional dairy products [10]. Studies have shown that *Lacticaseibacillus casei* and *lactobacillus coagulans* can improve the reproductive hormone level and antioxidant capacity in rats [11]. *Lacticaseibacillus casei* CGMCC1.8727 is now widely used in the food industry, mainly in the production of yoghurt and other dairy products. *Lacticaseibacillus casei* CGMCC1.8727 also has a variety of probiotic properties, especially in the treatment of animal intestinal diseases. Nevertheless, activities in the digestive tract make oral probiotics vulnerable to the adverse environment in the body, which includes gastric acid, digestive enzymes and bile acid salts, resulting in a decline or even loss of probiotic activity [12]. The unencapsulated *E. faeciumare* is very susceptible to simulated fish digestive conditions, according to present studies [13]. Microencapsulation technology embeds the core of the probiotic (solid, liquid or gas) in various wall materials, creating a dense barrier around the core to reduce its reaction with the external environment [14]. Microencapsulation of the core material can change the physical and chemical properties of the final product, increase the stability, avoid or reduce the inactivation of the core material, and prolong its storage time. Microcapsules can reduce the reaction between the core material and the external environment, thus improving the vitality of the substances in the capsule. Under appropriate conditions, the encapsulated active substances can be released quickly [15]. The research results of Eratte et al. [16] showed that microencapsulation technology can significantly improve the survival and competitiveness of *Lacticaseibacillus casei* CGMCC1.8727 in adhering to the intestinal wall and villi, so it can become an intestinal mucosal particle delivery system with no effect on its function.

In the context of alternatives to traditional antibiotics, antimicrobial peptides (AMPs) have become promising products due to their unique advantages. AMPs achieve their antibacterial effect by improving membrane permeability, inhibiting metabolism, and adjusting immune responses [17]. Furthermore, AMPs do not easily create drug resistance. AMPs are expected to supplement small–molecule antibiotics to fight multi–drug–resistant microorganisms in the future [18]. Since the discovery of natural AMPs that can widely attack a variety of pathogens, peptide–based therapy has been developed [19]. Bomidin is a recombinant AMP derived from BMAP–27 (a cathelicidin–derived AMP) via isolation and purification after expression in an *E. coli* expression system. Its relative molecular weight is 24.75 kDa [20]. It has been discovered that bomidin has been widely used to treat bacterial infections on the surface of skin and mucosa with characteristics of high antibacterial activity, small relative molecular weight, and weak haemolytic activity [21].

In this study, bomidin was combined with probiotics to treat gastrointestinal inflammation caused by *Vibrio parahaemolyticus*, and the effect was tested in vivo and in vitro, which would provide some reference for the treatment of gastrointestinal diseases.

## 2. Materials and Methods

### 2.1. Bacterial Strains and PCR Identification

The probiotic strain, *Lacticaseibacillus casei* CGMCC1.8727, was purchased from China’s general microbial strain preservation and management center. The pathogenic strain was *Vibrio parahaemolyticus* RIMD 2,210,663 strain, which was preserved by the zoonosis Laboratory of Nanjing Agricultural University.

Bacterial 16S rDNA universal primers were used for strain identification, and the primers were synthesized by Tsingke Biotechnology Company (Qingdao, China).

The 16srDNA gene–based identification was carried out using universal PCR primers 16S–F (5′-AGAGTTTGATCCTGGCTCAG-3′) and 16S–R (5′-GGTTACCTTGTTACGACTT-3′). Polymerase chain reaction (PCR) was performed using 25 µL contain 1 µL forward primer, 1 µL reverse primer, 12.5 µL Green Taq Mix, 9.5 µL ddH_2_O and 1 µL DNA template. The thermal cycler was setup for 30 cycles: initial denaturation at 94 °C for 5 min, denaturation at 94 °C for 30 s, annealing at 55 °C for 30 s, extension at 72 °C for 2 min, and a final extension at 72 °C for 10 min. The electrophoresis gel was run for verification at 1% agarose.

### 2.2. Preparation of Microcapsules

According to Songyajuan’s improved preparation process [22], the microcapsule preparation process was as follows: The activated *Lacticaseibacillus casei* CGMCC1.8727 was centrifuged at 4 °C at low temperature, the bacteria were collected (6000 r/min, 15 min), washed twice with 0.9% sterile normal saline, and then centrifuged again; the bacterial sludge was collected, and the bacterial sludge of *Lacticaseibacillus casei* CGMCC 1.8727 was diluted and resuspended with sterile normal saline to prepare a bacterial suspension. The appropriate proportion of gellan gum xanthan gum mixed solution was cooled after being sterilized at 110 °C for 10 min, and the bacterial suspension and mixed gum solution were evenly mixed. The mixed solution was dropped drop by drop into the chitosan solution of a certain concentration dissolved in 1% glacial acetic acid slowly stirred on the magnetic stirrer with a 5 mL disposable sterile medical syringe. The needle was about 5 cm from the liquid level of the solution. After the wet capsule was fully complexed, it was filtered with a screen. The required microcapsules of gellan gum xanthan gum chitosan *Lacticaseibacillus casei* CGMCC1.8727 were obtained by washing three times with sterile normal saline. The initial conditions for the preparation of *Lacticaseibacillus casei* CGMCC1.8727 microcapsules were as follows: chitosan concentration 0.6%, pH 5.5, mixed gum concentration of 1%, gellan gum and xanthan gum ratio of 1:4, bacterial suspension and mixed gum volume ratio of 1:5, bacterial gum and chitosan volume ratio of 1:4, and stirred for 40 min.

The viable number of *Lacticaseibacillus casei* CGMCC1.8727 was determined by plate colony counting method. A total of 1 g microcapsules was placed in 10 mL lysis solution, the capsules were exocytosed on a 180 rpm shaker for 30 min at 37 °C, diluted with 10 times gradient of sterile PBS buffer and vortically mixed, then coated on MRS AGAR medium. After 48 h of culture in a carbon dioxide incubator, plates with colony counts of 30–300 were selected and the average was calculated.

Embedding yield (%) = N/N_0_ × 100%

N: Total viable bacteria in microcapsules; N_0_: total number of live bacteria in the original bacteria solution before embedding.

### 2.3. Optimization of the Preparation Conditions

The morphology of microcapsules was measured with vernier caliper with a particle size of 80 microcapsules. In order to investigate the effects of seven factors including chitosan concentration, chitosan pH, the ratio of gellan gum and xanthan gum, the ratio of bacterial suspension to mixed colloid, the ratio of bacterial gel to chitosan, the concentration of mixed gum and stirring time on microencapsulated *Lacticaseibacillus casei* CGMCC1.8727, the viable number of microcapsules and the embedding yield were used as the indexes. The test was repeated three times. All factor levels were set based on references and pretests.

On the basis of a single factor test, an orthogonal test was carried out to explore the optimal process conditions for the preparation of microcapsules. Seven main factors affecting the preparation process were selected, and the orthogonal table of seven factors and four levels was designed by using IBM SPSS statistics software V26. See Table 1 for the orthogonal test design. The test was repeated three times.

Each factor level was set as follows. A: chitosan concentration: 0.3%, 0.6%, 0.9% and 1.2%; B: chitosan pH: 4.5, 5.0, 5.5 and 6.0; C: ratio of gellan gum to xanthan gum: 2:3, 1:4, 1:6 and 1:8; D: volume ratio of bacteria suspension to mixed gel: 1:5, 1:10, 1:15 and 1:20; E: volume ratio of agar to chitosan: 1:2, 1:4, 1:6 and 1:8; F: mixing time: 30 min, 40 min, 50 min and 60 min; G: mixed glue concentration: 0.6%, 0.8%, 1.0% and 1.2%.

### 2.4. Tolerance Test of Microencapsulated Lacticaseibacillus casei CGMCC1.8727

In order to study the effect of gastric juice and bile salt on free and microencapsulated *Lacticaseibacillus casei* CGMCC1.8727, microencapsulated samples and *Lacticaseibacillus casei* CGMCC1.8727 suspension samples were exposed to simulated gastric juice and bile salt.

One gram of microencapsulated *Lacticaseibacillus casei* CGMCC1.8727 was placed in 10 mL artificial gastric juice at 37 °C, and 5 treatment times were set as 0 h, 0.5 h, 1 h, 1.5 h and 2 h. After treatment according to the above time period, the microcapsules were filtered, collected, and washed, and then put into 10 mL of unpacking solution for unpacking. After the completion of unpacking, the number of live bacteria was measured and calculated respectively. In addition, 1 mL free bacterial suspension was used as the control, and the volume of the original bacterial suspension used in the preparation of the bacterial suspension was the same as that used in the microcapsule. The specific content of the simulated bile salt test is the same as that of the simulated gastric juice test.

One gram of *Lacticaseibacillus casei CGMCC1.8727* microcapsules was placed in 10 mL artificial intestinal fluid at 37 °C, and the samples were shaken at 180 r/min in a constant temperature shaker at 37 °C. Four treatment periods were set, including 0.5 h, 1 h, 1.5 h, and 2 h, respectively. The number of viable bacteria dissolved from the microcapsules was measured and calculated.

Three different batches of microencapsulated probiotics were packed into sterile centrifuge tubes and stored at 4 °C and room temperature respectively. Take an appropriate amount of microcapsule samples stored for 1 d, 3 d, 7 d, 14 d, 21 d and 28 d, and detect the number of viable bacteria in the microcapsule after crushing in the solution. The free bacterial suspension was used as control.

### 2.5. In Vitro Test for Antibacterial Activity

According to studies, the antibacterial activity of *Lacticaseibacillus casei* CGMCC1.8727 was determined by the Oxford cup method [23]. The growth curve of *Vibrio parahaemolyticus* RIMD2210663 was measured by an automatic growth curve analyzer (BioSan, Riga, Latvia). The growth curve was determined by at least three independent experiments. Minimum Inhibitory Concentration (MIC) Test for Antimicrobial Peptides was measured by automatic microplate reader (TACAN, Switzerland) according to the standards of Clinical and Laboratory Standards Institute (CLSI).

### 2.6. In Vivo Test for Antibacterial Activity

The experimental animals used in this study were all SPF–grade female Sprague Dawley rats from Vital River Laboratory Animal Technology Company (Beijing, China). The rats were 7 weeks old and weighed 190–210 g at the time of experiments. All rats were kept separately in a specific pathogen–free cage in the experimental animal center of Nanjing Agricultural University. The temperature and humidity shall be controlled at 21~23 °C and 60~65% respectively. All rats were randomly divided into seven groups: negative control group, positive control group, free probiotics group, microencapsulated probiotics group, antibacterial peptide group, antibacterial peptide + microcapsule interval of 6 h group and antibacterial peptide + microcapsule interval of 9 h group, with 10 rats in each group. The rats were treated with drugs and the status was observed. Two days before the experiment, 5 g/L streptomycin was fed to the rats for 24 h to remove intestinal microbiota. The day before the experiment, the rats were deprived of food and water for 24 h. On the day of the experiment, each rat was first neutralized with 1 mL 5 % NaHCO_3_ in advance. After 20 min, rats in a normal saline group (positive group), naked bacteria group (YL group), microcapsule group (WN group), antibacterial peptide group (KJT group), antibacterial peptide + microcapsule interval 6 h group (6 h group) and antibacterial peptide + microcapsule interval 9 h group (9 h group) were gavaged with 2 mL (1 mL/100 g: weight) of *Vibrio parahaemolyticus* respectively, and rats in normal control group (negative group) were gavaged with 2 mL PBS. The next day, the treatment was started 2 h after the same amount of bacteria was administered. The naked bacteria group was given 2 mL of free *Lacticaseibacillus casei* CGMCC1.8727 suspension with a concentration of 10^9^ CFU/mL. The microcapsule group was given 2 mL probiotic microcapsule suspension at a dose of 10^9^ CFU/mL. The normal saline group was given 2 mL of normal saline by gavage. The two combined medication groups were given antibacterial peptides by gavage, and then an appropriate amount of microcapsules were given by gavage at intervals of 6 h and 9 h respectively. The rats were closely observed during the treatment.

The mental state, feeding state, and clinical manifestations of the rats during the administration and recovery were observed and recorded. During the administration period, the rats were dissected every 1, 2, 3, 6, and 10 days in each group, and 1 g of intestinal tissue was taken and ground to obtain the supernatant, and the changes of inflammatory factors (TNF–α, IL–1β, IL–6, IL–8) in the supernatant were detected by ELISA kit. At the same time, the intestinal tissues of rats were fixed with 4% paraformaldehyde buffer, and pathological sections were made. Using Image Pro Plus 6.0 analysis software, the height and recess depth of five intact villi in each slice were measured in millimetres, and the ratio of villi height to recess depth was calculated. Fecal samples were collected in each experimental group and sent to Sangon Biotech (Shanghai, China) Company for metagenomic sequencing to detect the changes in intestinal flora before and after administration. To detect the concentration of Vibrio parahaemolyticus in feces after drug administration, the feces were soaked in PBS for a certain time, and an appropriate amount of samples were diluted in PBS and coated on TCBS selective medium. The samples were incubated in an anaerobic incubator at 37 °C for 18 h, and the green colonies were counted separately in plates. 16S primers were designed and identified to determine whether the colony was *Vibrio parahaemolyticus* RIMD 2,210,663 in combination with primers specific for its virulence gene *tdh/tlh*. The specific sequences of primers used in the test were shown in Table 2. The primers were synthesized by Tsingke Biotechnology Company (Qingdao, China).

### 2.7. Statistics

Prism 8 (GraphPad) was used to compare and analyze the data of each experimental group of rats. Statistical significance was set at *p* < 0.05. The data was displayed as mean ± SD. All in vivo and in vitro experiments were independently repeated at least three times.

The paired–end reads obtained from the second–generation sequencing were first spliced according to the overlapping relationship, and then the sequence quality was controlled and filtered after the samples were distinguished, and then OTU (Operational Taxonomic Units) clustering analysis and species taxonomy analysis were carried out. According to OTU clustering analysis results, OTU can be analyzed for diversity index and sequencing depth; According to the taxonomic information, the statistical data of community structure can be analyzed at different classification levels. On the basis of the above data analysis, a series of analyses such as alpha diversity analysis and beta diversity analysis can be carried out on the community composition of biological multi–samples.

## 3. Results

### 3.1. Verification of Lacticaseibacillus casei CGMCC 1.8727

**Figure 1:**
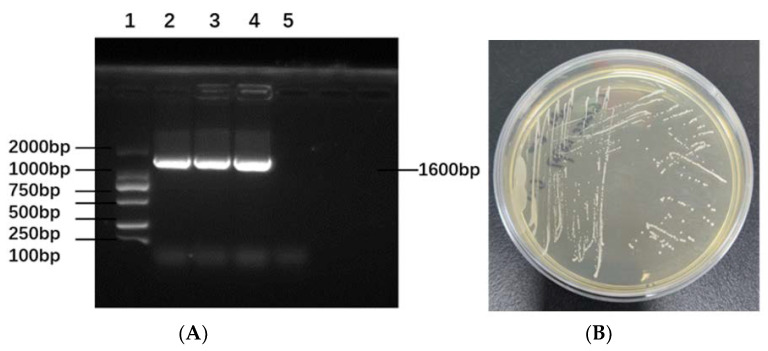
Verification of *Lacticaseibacillus casei CGMCC 1.8727*. (**A**) PCR identification results. 1: DL2000 DNA Marker 2–4: *Lacticaseibacillus casei* CGMCC1.8727 5: negative control; (**B**) Colony morphology of *Lacticaseibacillus casei* CGMCC1.8727.

The PCR identification results of *Lacticaseibacillus casei* CGMCC 1.8727 are shown in Figure 1A. The band size was 1600 bp. Using the PCR results along with morphological identification, the strain was determined to be *Lacticaseibacillus casei* CGMCC1.8727. The colony morphology of *Lacticaseibacillus casei* CGMCC 1.8727 is shown in Figure 1B.

### 3.2. Morphology of Microencapsulated Lacticaseibacillus casei CGMCC1.8727

**Figure 2:**
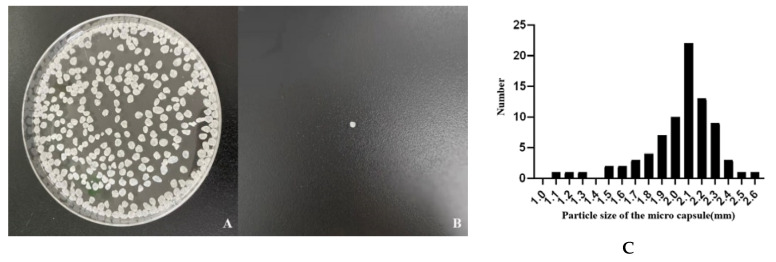
Image and particle size distribution of microcapsules. (**A**) The morphology of microcapsules; (**B**) Morphology of a single microcapsule; (**C**) Particle size distribution of microcapsules (*n* = 80).

Figure 2A,B shows that the sizes of the microencapsulated *Lacticaseibacillus casei* CGMCC1.8727 were uniform and the shapes were close to spherical. Figure 2C shows that the average particle size of the microcapsules was 2.04 ± 0.2743 mm.

### 3.3. Single–Factor Tests

**Figure 3:**
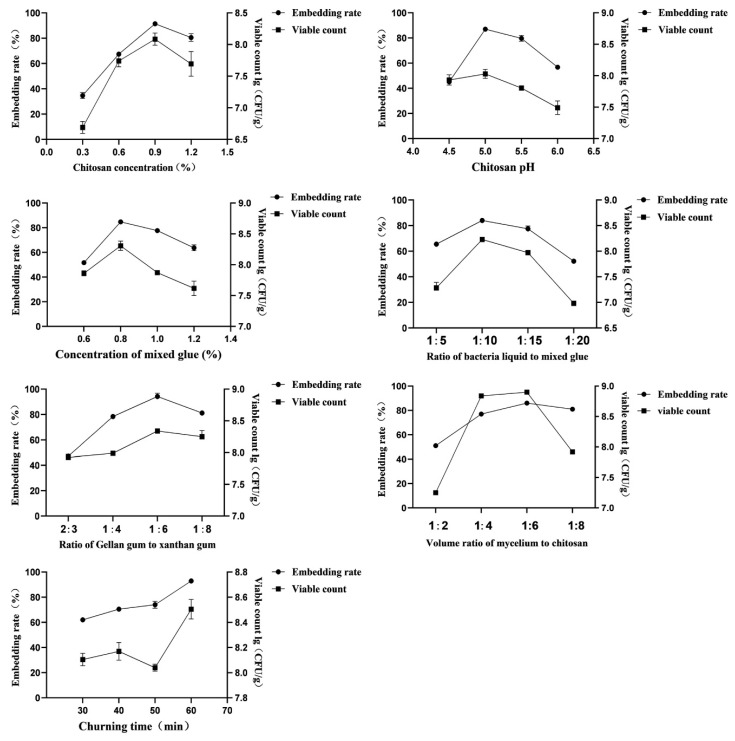
Seven influence factors on embedding rate and viable count of microcapsules.

It can be seen from Figure 3, when the concentration of chitosan was 0.9%, the pH 5.0 of chitosan, the concentration of mixed gel solution was 0.8%, the product ratio of bacterial suspension to mixed colloid was 1:10, the mass ratio of gellan gum to xanthan gum was 1:6, the volume ratio of mycolloid to chitosan solution was 1:6, and the stirring time was 60 min, the number of viable bacteria per unit mass and the embedding rate of the microcapsules were the highest.

### 3.4. Orthogonal Test

Table 3 shows the orthogonal test results for microencapsulated *Lacticaseibacillus casei* CGMCC1.8727. When the concentration of chitosan was 1.2%, the pH of chitosan was 4.5, the ratio of gellan gum to xanthan gum was 1:4, the volume ratio of bacterial suspension to mixed gum was 1:10, the volume ratio of bacterial gum to chitosan was 1:4, stirring time was 40 min and the concentration of mixed gum was 0.8% and the maximum encapsulation efficiency of *Lacticaseibacillus casei* CGMCC1.8727 microcapsules was 93.12 ± 1.44%. The microcapsules of *Lacticaseibacillus casei* CGMCC1.8727 were prepared with the above optimal parameters for the follow–up tests.

### 3.5. Stability Tests

#### 3.5.1. Stability of *Lacticaseibacillus casei* CGMCC1.8727 Microcapsules in Artificial Gastrointestinal Tract Environment

**Figure 4:**
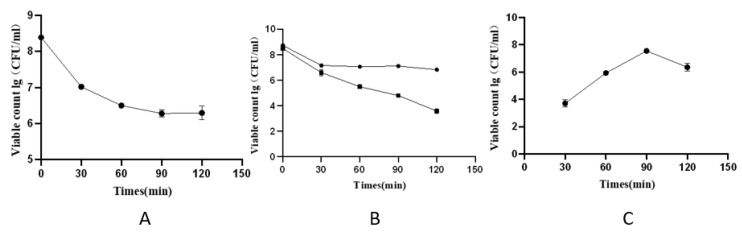
In vitro performance test of microcapsules. (**A**) Acid resistance test of microcapsules; (**B**) Bile salt tolerance test of microcapsules: the circular dots were the experimental group, and the square dots were the control group; (**C**) Enterolysis test of microcapsules.

After microencapsulated *Lacticaseibacillus casei* CGMCC1.8727 was treated with gastric juice, the number of viable bacteria gradually decreased with time due to the strongly acidic environment (Figure 4A). The number of living bacteria in the free probiotic decreased rapidly. When fre*e probiotic*s were treated in gastric juice for 30 min, a dilution of 10:4 was taken to measure the number of living bacteria, and no living bacteria were detected. Nevertheless, under the same conditions, the number of embedded living probiotics was significantly greater than that of non–embedded probiotics. After 120 min of treatment, the number of living bacteria was 6.3 ± 0.04 log CFU/mL.

After 120 min of treatment in the simulated bile salt solution, the number of viable packaged probiotics was significantly higher than that of probiotics in the free state (Figure 4B). The results showed that the number of viable bacteria was 6.83 ± 0.007 log CFU/mL, indicating bile salt tolerance.

The number of viable bacteria in the microcapsule reached the maximum when the probiotic microcapsule was treated with artificial intestinal juice for 90 min (Figure 4C). However, when the artificial intestinal juice treatment was continued for 90 min, the number of viable bacteria decreased. The results showed that when the probiotic microcapsules were treated with simulated intestinal juice for 90 min, the bacteria in the probiotic microcapsules were released completely and the microcapsules showed good enteric solubility. Over time, the number of living bacteria will decrease, and the released active probiotics will not adapt to the intestinal fluid environment, resulting in a slight decrease in activity.

#### 3.5.2. Stability of Microencapsulated *Lacticaseibacillus casei* CGMCC1.8727 during Storage

**Figure 5:**
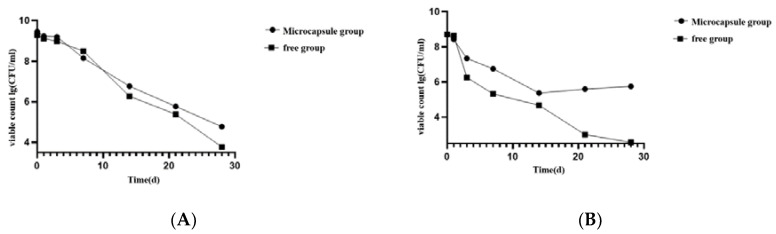
Microcapsule storage experiment. (**A**) Microcapsule storage experiment at room temperature; (**B**) Microcapsule storage experiment stored at 4 °C.

The activity of microencapsulated *Lacticaseibacillus casei* CGMCC1.8727 was best when preserved at 4 °C (Figure 5), and the number of viable bacteria *Lacticaseibacillus casei* CGMCC1.8727 was higher than that of the free probiotic solution, indicating that microencapsulated *Lacticaseibacillus casei* CGMCC1.8727 has good stability and probiotic activity at 4 °C. After 28 days of storage at 4 °C, the number of viable bacteria remained at 5.87 ± 0.15 log CFU/mL.

#### 3.5.3. Oxford Cup Bacteriostasis Test for *Lacticaseibacillus casei* CGMCC1.8727

**Figure 6:**
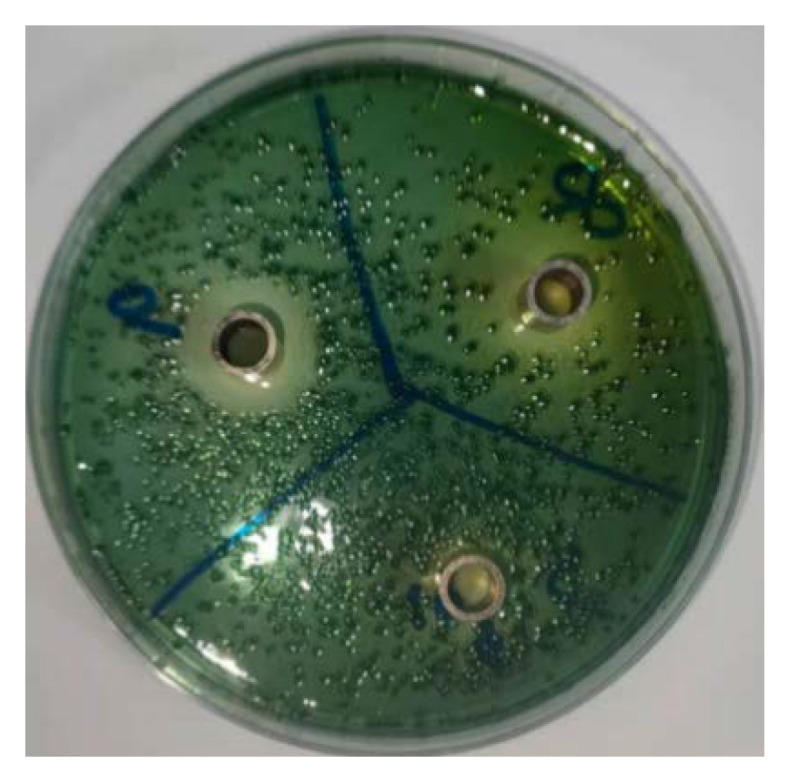
Oxford Cup bacteriostatic test.

The criteria for the oxford cup bacteriostasis test were as follows: diameter of bacteriostatic circle > 20 mm, extreme sensitivity; diameter of the bacteriostatic circle between 15 and 20 mm, high sensitivity; diameter of the bacteriostatic circle between 10 and 14 mm, moderate sensitivity; diameter of bacteriostatic circle < 10 mm, low sensitivity; diameter of bacteriostatic circle is 0 mm, no sensitivity. The results showed that *Lacticaseibacillus casei* CGMCC1.8727 had a good inhibitory effect on *Vibrio parahaemolyticus* RIMD2210663 (Table 4 and Figure 6).

### 3.6. In Vitro Experiments

**Figure 7:**
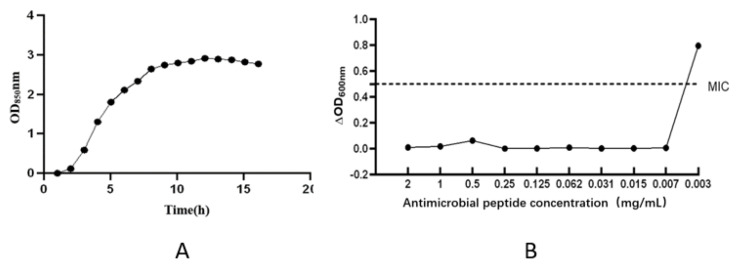
Determination of growth curve of *Vibrio parahaemolyticus* and minimum inhibitory concentration (MIC) test for antimicrobial peptides. (**A**) Determination of growth curve of *Vibrio parahaemolyticus*; (**B**) Minimum inhibitory concentration (MIC) test for antimicrobial peptides.

Figure 7A shows the growth curve of *Vibrio parahaemolyticus* cultured overnight. As shown in the figure, the OD_850nm_ of *Vibrio parahaemolyticus* remained generally unchanged within 0–1 h. The concentration increased rapidly between 1 and 9 h and reached its peak at 9 h. The OD_850_ nm remained at 2.74 after the time point of 9 h. The figure shows that the retardation period of *Vibrio parahaemolyticus* was 0–1 h, the growth logarithmic period was 1–9 h and the stable period was reached after 9 h. Figure 7B shows the results of the 96–well plate experiment. It was observed by the naked eye that there was no obvious bacterial precipitation in wells 1–9, and there was bacterial precipitation in wells 10 and 11. The results of the experiment repeated three times were the same. The above results show that the MIC of the antibacterial peptide was 0.007 mg/mL. The OD_600nm_ value decreased with an increase in bomidin concentration. Bomidin has obvious antibacterial activity against *Vibrio parahaemolyticus* and good bactericidal effect.

### 3.7. In Vivo Test Index Results and Analysis

#### 3.7.1. Analysis of Mental State and Survival Curve of Rats

**Figure 8:**
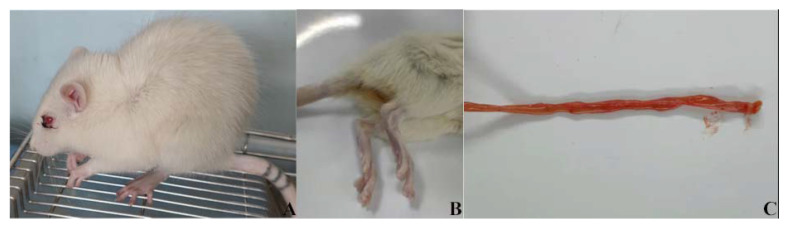

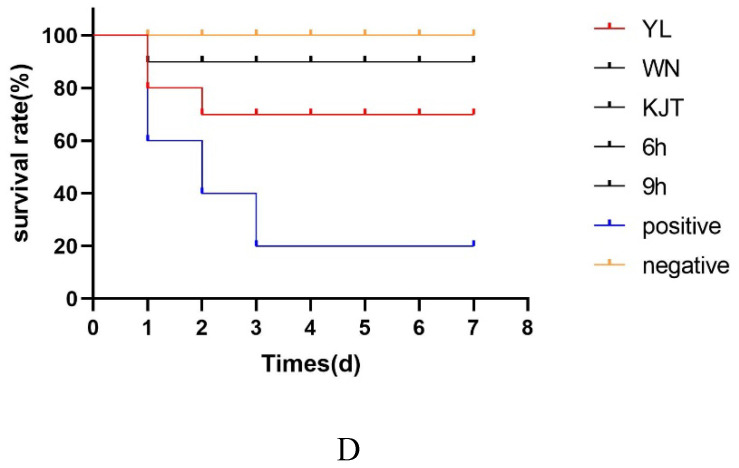
Symptom, lesion and survival curve of rats. (**A**) External symptoms of rats in the positive group; (**B**) Severe diarrhea in dead rats; (**C**) Intestinal lesions after dissection in rats; (**D**) Survival curve: YL group: naked bacteria group; WN group: microcapsule group; KJT group: antibacterial peptide group; 6 h group: antibacterial peptide + microcapsule interval 6 h group; 9 h group: antibacterial peptide + microcapsule interval 9 h group; positive group: rats in a normal saline group; negative group: the control group (*n* = 10).

In the positive group, the rats were depressed, crouching, trembling, secretions in the eyes, acute attacks, abdominal pain, diarrhea, and yellow watery stool, intestinal bleeding, and death in severe cases (Figure 8A–C). The survival curve for the rats in the seven groups is shown in Figure 8D (*p* < 0.05). Four rats in the positive control group died on the first day of the experiment, and two rats died on the second and third days, respectively. In the free probiotics group, two died on the first day and one died on the second day. One rat died on the first day in the other experimental groups. There was no death in the later period. The rats in the negative control group were in good condition during the test period. The results showed that the cure rate of the free probiotics group was 62.5%, and that of the microcapsule group, the antibacterial peptide group, and the combined drug group were all 87.5%.

#### 3.7.2. Analysis of Pathological Sections from Rat Duodenum

Table 5 shows the ratio of villi height to recess depth in each group. The results show that after treatment, the ratio of villi height to recess depth in the microcapsule group was significantly higher than that in the free probiotics group (*p* < 0.05). There was no significant difference between the free probiotics group and the antibacterial peptide group (*p* > 0.05). On the ninth day after treatment, the ratio of villi height to recess depth in the combined group was significantly higher than that in the free probiotics group.

**Figure 9:**
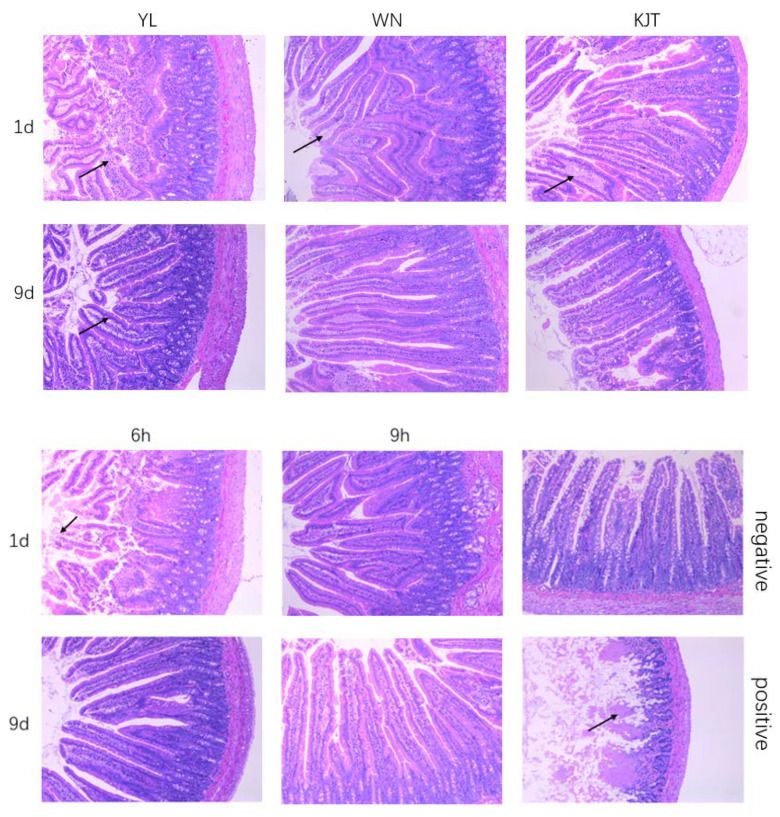
HE stained sections of the duodenum of rats. The magnification of all pathological sections was 100. YL group: naked bacteria group; WN group: microcapsule group; KJT group: antibacterial peptide group; 6 h group: antibacterial peptide + microcapsule interval 6 h group; 9 h group: anti–bacterial peptide + microcapsule interval 9 h group; positive group: rats in a normal saline group; negative group: the control group.

Figure 9 shows the observation results of pathological sections of the rat intestinal tract stained with HE. In the field of vision of the negative control group, it can be seen that the structure of each layer was clear, the mucosal epithelium was complete, the number of intestinal glands was rich and closely arranged and the myocytes in the muscular layer were closely arranged. In the positive group, a large number of intestinal villi were necrotic and dissolved, showing irregular eosinophilic substances. In the YL group, a large number of intestinal villous epithelium and lamina propria were separated in the field of vision on the first day of treatment, whereas epithelial cell necrosis and abscission, oedema of lamina propria, eosinophils and a high level of cell necrosis and nuclear fragmentation were seen in the field of vision on the ninth day. In the WN group, a large number of intestinal villous epithelium were separated from the lamina propria on the first day and a high level of oedema of lamina propria and eosinophilic substances could be seen. On the ninth day, a low level of necrosis was observed in the epithelial cells as well as abscission. In the KJT group, a large area of erosion, necrosis and abscission of a large number of intestinal epithelial cells and oedema of a large number of intestinal villi lamina propria and eosinophils were seen in the field of vision on the first day. A small amount of necrosis and abscission of epithelial cells were seen in the field of vision on the ninth day. In the 6 h interval group, a large number of intestinal villi epithelial necrosis and abscission could be seen in the field of vision on the first day after treatment, and a small amount of epithelial cell necrosis and abscission could be seen in the field of vision on the ninth day after treatment. No obvious pathological changes were found on the first and ninth days after treatment in the group with an interval of 9 h. The results showed that microencapsulated probiotics could effectively alleviate the intestinal villi damage caused by *Vibrio parahaemolyticus* colonization and that the combination of drugs could accelerate intestinal recovery.

#### 3.7.3. Changes in Inflammatory Factors in Duodenal Tissue Supernatant

**Figure 10:**
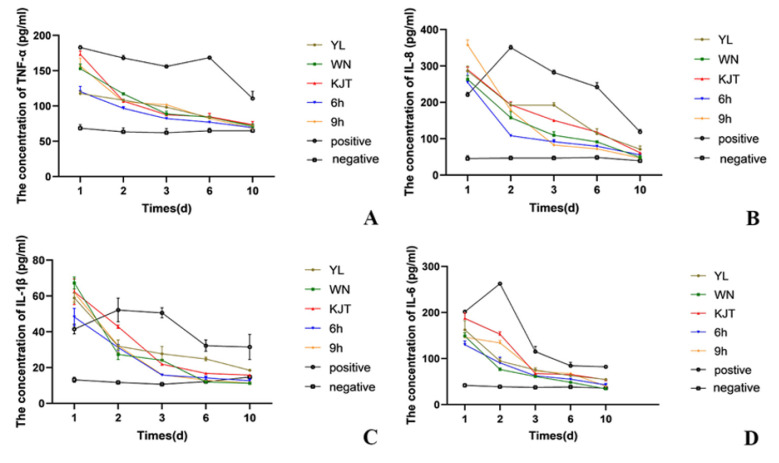
Changes of inflammatory factor concentration in the supernatant of duodenal tissue. (**A**) The changes of TNF–α; (**B**) The changes of IL–8; (**C**) The changes of IL–1β; (**D**) The changes of IL–6. YL group: naked bacteria group; WN group: microcapsule group; KJT group: antibacterial pep–tide group; 6 h group: antibacterial peptide + microcapsule interval 6 h group; 9 h group: antibacterial peptide + microcapsule interval 9 h group; positive group: rats in a normal saline group; negative group: the control group (*n* = 3).

Figure 10A shows TNF–α in the supernatant of rat duodenal tissue within 10 days after treatment (*p* < 0.05). The expression level of TNF–α in the negative control group was maintained at around 60–70 pg/mL. The expression level of TNF–α in all experimental groups on the first day was significantly higher than that in the negative control group and decreased the next day, but the TNF–α expression in the microencapsulated probiotics group decreased after the first day, and the rate of decline in the later period was faster than that in the free probiotics group, which was closer to the normal level. The expression of TNF–α in the antibacterial peptide group was slightly faster than that in the free probiotics group, but there was no significant difference between the two groups. In the combination group, the decrease rate of the TNF–α expression level was faster than that in both the microcapsule group and the experimental group. An interval of 9 h showed a greater decrease, and the curative effect was better. The changes were similar to those of IL–6, there was no significant difference between the 6 h group and the 9 h group (Figure 10B–D). These results indicate that the therapeutic effect of microencapsulated probiotics was superior to that of free probiotics, and the addition of AMPs can improve the therapeutic effect. The data are shown in Table 6.

#### 3.7.4. Concentration Change and Colony Identification of *Vibrio parahaemolyticus* in Rat Faeces

**Figure 11:**
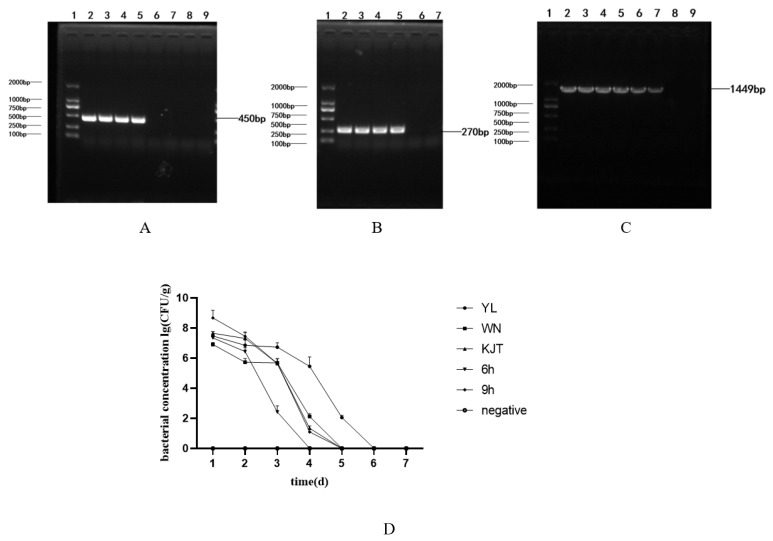
Concentration changes and colony identification of *Vibrio parahaemolyticus* in feces. (**A**) 1: DL2000 DNA Marker 2–5: *Vibrio parahemolyticus* RIMD2210663 *tlh* virulence gene 6–9: negative control; (**B**) 1: DL2000 DNA Marker 2–5: *vibrio parahemolyticus* RIMD2210663 *tdh* virulence gene 6,7: negative control; (**C**) 1: DL2000 DNA Marker 2–7: *Vibrio parahaemolyticus* RIMD2210663 16SrRNA 8,9: negative control; (**D**) The variation of *Vibrio parahaemolyticus* RIMD 2,210,663 concentration in feces: YL group: naked bacteria group; WN group: microcapsule group; KJT group: antibacterial peptide group; 6 h group: antibacterial peptide + microcapsule interval 6 h group; 9 h group: antibacterial peptide + microcapsule interval 9 h group; negative group: the control group (*n* = 3).

The bacteria in rat feces were identified as *Vibrio parahaemolyticus* via PCR (Figure 11A–C). The changes in the concentration of *Vibrio parahaemolyticus* in rat feces within 1 week after administration were shown in Figure 11D (*p* < 0.05). The concentration of *Vibrio parahaemolyticus* in 1 g of feces from the microencapsulated probiotics group was lower than that in the free probiotics group, and *Vibrio parahaemolyticus* could not be detected on the fifth day. However, *Vibrio parahaemolyticus* could be detected in the free probiotic group for six consecutive days, and the bacterial concentration in the first four days was higher than that in the microcapsule group. The removal of *Vibrio parahaemolyticus* was slightly faster in the antibacterial peptide group than in the microcapsule group, but there was no significant difference between the two groups. No bacteria were detected on the fourth day in the group with an interval of 6 H, indicating that the antibacterial effect of the combination of bomidin with *Lacticaseibacillus casei* CGMCC1.8727 microcapsules was better than microcapsules alone. The above results showed that the microencapsulated probiotics had a good effect on the removal of *Vibrio parahaemolyticus* in rats, and the combination of bomidin with microencapsulated *Lacticaseibacillus casei* CGMCC1.8727 could remove bacteria faster than microencapsulated *Lacticaseibacillus casei* CGMCC1.8727 alone. The data are shown in Table 7.

### 3.8. Analysis of Macrogenomic Sequencing Results of Rat Faeces in Each Experimental Group

#### 3.8.1. Effective Sequence Analysis

Through macrogenomic sequencing of the v3–v4 region of the 16S rDNA gene in the feces of rats in each experimental group, a total of 734,241 high–quality optimized sequences were obtained. The average number of sequences in each sample was 61,186, and the average sequence length was 418 bp; the length of these sequences was generally more than 350 bp (Table 8). The sequencing depth of this study tended to saturation. Cluster analysis of OTUs was carried out using the USEARCH (Ultra–fast sequence analysis) sequence alignment method. A sequence similarity of 97% was used as the OTU classification threshold. The sequence with the highest abundance in each OTU was defined as the representative sequence of the OTU, and the microbial community identification results of each sample at each classification level were analysed.

#### 3.8.2. Alpha Diversity Analysis among Intestinal Microflora of Rats in Experimental Groups

Alpha diversity refers to the diversity within a specific region or ecosystem and reflects the richness and evenness of microbial communities. Commonly used indicators to measure alpha diversity include the ACE index, Chao1 index, Simpson index, and the Shannon index. The Shannon index comprehensively considers the richness and evenness of a community. The higher the Shannon index, the higher the diversity of the community. The Shannon index is more sensitive to community richness and rare OTU compared with the other indicators. The larger the Chao1 or ACE index, the higher the richness of the community, whereas for the Simpson index, the opposite is true (the higher the Simpson index, the lower the community diversity). The Shannon even index is used to reflect the evenness of distribution of an individual number of species in the community. The coverage index indicates that this result can reflect the real situation of microorganisms in a sample.

Table 9 shows the alpha diversity index of rat fecal flora under each treatment. Comparing the alpha diversity index of feces from the first day and feces from the ninth day after treatment in each group, it was found that the Shannon index of the free probiotics group decreased, indicating that the community diversity decreased. The Shannon index for the other groups increased, with the microcapsule group showing the highest increase (*p* < 0.05); the community diversity was also highest in this group. The Chao index and ACE index were shown to be decreased in the 9 h interval group; however, in the 6 h interval group, the Chao index increased. The Chao index also increased in the other groups, but the increase in the free probiotics group was smaller than that observed in the microcapsule group. Compared with the ninth day after treatment, the Simpson index in the free probiotics group was increased, indicating that the community diversity decreased. In the other groups, the Simpson index decreased, indicating an increase in diversity, whereas the Simpson index was most decreased in the microcapsule group (*p* < 0.05) and the community diversity was the highest. The combined application of microcapsules and AMPs for 6 h effectively improved the species diversity and richness of the intestinal microbiota, whereas the population richness and diversity in the faeces from the free probiotics group were significantly lower compared with that from the microcapsules group.

#### 3.8.3. Beta Diversity Analysis of Intestinal Microbiota of Rats in Experimental Groups

**Figure 12:**
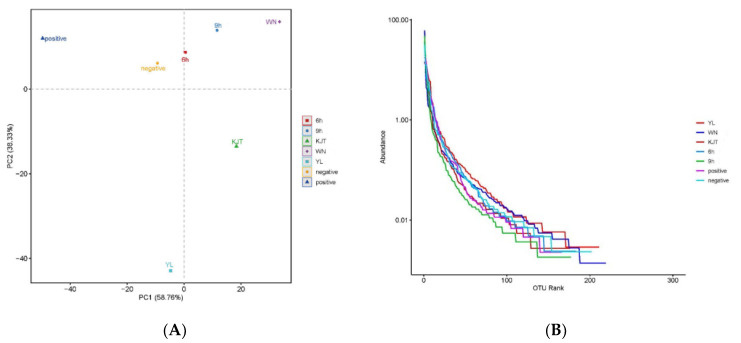
Beta diversity analysis of intestinal microbiota of rats in experimental groups. (**A**) PCA analysis of intestinal microbiota structure among experimental rats in each group; (**B**) Rank abundance curve. YL group: naked bacteria group; WN group: microcapsule group; KJT group:antibacterial pep–tide group; 6 h group: antibacterial peptide + microcapsule interval 6 h group; 9 h group: anti–bacterial peptide + microcapsule interval 9 h group; positive group: rats in a normal saline group; negative group: the control group.

Beta diversity refers to the comparison of diversity among various ecosystems and reflects the change rate of species composition along the environmental gradient or between communities. It is generally used to describe the response of biological species to environmental heterogeneity. In this study, the differences between the microbial community structures in the intestines of experimental rats in each group were compared via PCA. The closer the sample species composition, the smaller the distance in the PCA diagram. Figure 12A shows that the microbial community structures of the positive group and the normal group were significantly different, the microbial community structures of the interval medication and the microcapsule groups were significantly recovered to close to that of the normal group and the difference between the free probiotics group and the normal group was significant. The results showed that 6 h interval administration could better restore the structure of the fecal flora to near–normal levels.

The rank abundance curve is another method for analyzing diversity. To construct the curve, the number of sequences contained in each OTU in a single sample was counted, and then, the OTUs were ranked according to abundance. The OTU level was recorded on the horizontal axis, and the number of sequences contained in each OTU (or the relative percentage content of the sequence number in the OTU) was recorded on the vertical axis. The rank abundance curve is used to explain two aspects of sample diversity at the same time: the richness and the evenness of species contained in the sample. The length of the curve on the horizontal axis reflects the species richness; the wider the curve, the richer the species composition. The height of the curve reflects the evenness of species composition; the flatter the curve, the more uniform the species composition. Figure 12B shows that the microcapsule group had the highest richness and evenness in species composition amongst all the groups tested.

#### 3.8.4. Changes in Composition and Structure of Rat Faecal Flora at the Phylum and Genus Level

**Figure 13:**
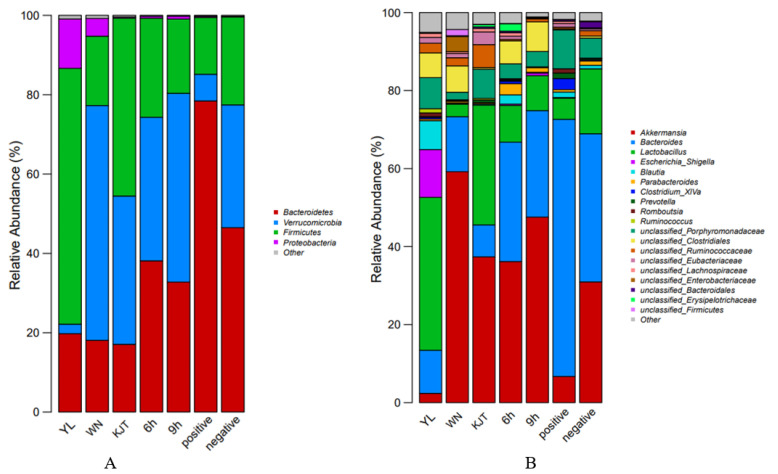
Histogram of relative abundance of fecal flora in each group at phylum and genus level. (**A**) Colony distribution at the phylum level; (**B**) Colony distribution at the genus level. YL group: naked bacteria group; WN group: microcapsule group; KJT group: antibacterial pep–tide group; 6 h group: antibacterial peptide + microcapsule interval 6 h group; 9 h group: antibacterial peptide + microcapsule interval 9 h group; positive group: rats in a normal saline group; negative group: the control group.

Based on the OTU classification results, the microbial flora and its composition structure in the feces of rats in the test groups were analyzed. The dominant phyla in the feces of rats in all test groups were Firmicutes, Bacteroidetes, Verrucomicrobia, and Proteobacteria, accounting for more than 98% of the total community. Through OTU abundance analysis, the species with the highest rank and the highest abundance at the gate level were selected to obtain the relative abundance map of each group of samples and to observe and compare which species had higher abundance as well as their proportions and change trends. Figure 13A shows that Bacteroidetes and Verrucomicrobia were the main phyla in the microflora in the intestines of rats in the negative control group, as can be seen from the variation trend of microbial flora in each treatment group. The abundance of Bacteroidetes increased sharply, whereas the abundance of Verrucomicrobia decreased sharply in the positive group after the challenge. The abundance of Bacteroidetes decreased sharply in the faeces of rats in each group after the administration of probiotics, whereas the abundance of Verrucomicrobia increased significantly, and the number of *Proteus* Bacteroidetes in the free probiotics group increased sharply. As can be seen from Figure 13B, in the distribution of colony composition, the 6 h and 9 h experimental groups were closer to the normal negative rat combination. Although the number of lactic acid bacteria in the free group increased somewhat, the number of Escherichia coli also increased sharply. Therefore, the effect of the combined drug was better than that of the single drug.

## 4. Discussion

The issue of antibiotic resistance has raised great concern across the world, and its harm is becoming increasingly serious. Both probiotics and antimicrobial peptides are hot research areas. Moreover, the discovery and study of natural anionic polysaccharides and chitosan can improve the stability of probiotics in the gastrointestinal tract. *Lacticaseibacillus casei* CGMCC1.8727 was embedded by microencapsulation technology using two kinds of mixed natural anionic polysaccharides and chitosan as carriers. Compared with free probiotics, microencapsulation significantly enhanced the growth activity and storage stability of probiotics under simulated gastrointestinal conditions, demonstrating that the combination of Bomidin and probiotics could effectively improve its therapeutic effect, and has a better recovery effect after treatment.

Xanthan gum is a natural polymer that can be obtained from bacteria in the genus *Xanthomonas* and is widely used in the food and drug industry. Due to its physical and chemical properties, xanthan gum has been used in the development and improvement of drug delivery systems [24]. This biomaterial has many advantages, including good biocompatibility, good biodegradability, non–toxicity in nature, and good physical stability in the presence of cations [25]. Gellan gum is an anionic extracellular polysaccharide with good biocompatibility and non–toxicity that forms a gel in the presence of cations. Gellan gum has been developed for use in a variety of physical and chemical gel processes, including hydrogels, nano–hydrogels, beads, films, and patches, with adjustable mechanical and physical, and chemical properties.

Extrusion, emulsification, and polymerisation are the most commonly used technologies and methods for encapsulating microorganisms [26]. The extrusion method is simple, inexpensive, and has a high bacterial survival rate [27]. In this study, chitosan and two mixed natural anionic polysaccharides were used to embed *Lacticaseibacillus casei* CGMCC1.8727 in microcapsules. Microcapsules with high probiotic activity were prepared by extrusion technology. The reaction mechanism involves chitosan dissolved in an aqueous acid solution. Chitosan’s positively charged amino group in an acidic solution and negatively charged anionic polysaccharide mixture form a co–gel material via a polyelectrolyte complex. This gel was used as a microcapsule wall material to protect the probiotics from the impact of the external environment. The results of single–factor and orthogonal tests showed that the highest encapsulation efficiency of microencapsulated probiotics was 93.12 ± 1.44%, and the size was 2.04 ± 0.2743 mm.

Based on the preparation of *Lacticaseibacillus casei* CGMCC1.8727 microcapsules with a good embedding effect, the in vitro characteristics of the probiotic microcapsules were studied. Through a simulated gastric acid resistance test, a simulated bile salt tolerance test, and a simulated intestinal fluid release test, the difference in the number of living bacteria after treatment with free probiotics and microencapsulated probiotics was compared with test the effect of microencapsulation. Finally, a storage stability test and an in vitro bacteriostasis test were conducted. The test results showed that the free probiotics could not tolerate the low–pH gastric juice environment. However, the microencapsulated *Lacticaseibacillus casei* CGMCC1.8727 tolerated these conditions well, and a higher number of live bacteria were retained under the protection of two natural anions and chitosan. In the bile salt tolerance test, the exposed probiotics were extremely sensitive to the bile salt environment, and the number of living bacteria decreased sharply. Studies have shown that the viability of free *lactobacillus johnsonii* ATCC33200 decreases rapidly when exposed to simulated bile salt solution, and it is highly sensitive to bile salt and lipase [28]. Compared with the free probiotics, the microencapsulated *probiotics* tolerated the bile salt conditions well. Microencapsulated probiotics must be released into the intestinal environment as soon as possible and then adsorbed and colonized after reaching the intestinal mucosa so they can play their prebiotic role. In the artificial intestinal juice test, we detected that microencapsulated *Lacticaseibacillus casei* CGMCC1.8727 was released in approximately 90 min, indicating that the enteric digestion effect was good. After continuous digestion for 6 h, the final viable bacterial count of *Lactobacillus rhamnose*–F4 GG beads prepared by Kawai Lai [29] exceeded 9 lg (CFU/mL). The results of the storage stability test for the microencapsulated probiotics showed that encapsulated *Lacticaseibacillus casei* CGMCC1.8727 had better stability than free probiotics and that the effect was better at lower temperatures. The results of the Oxford cup bacteriostasis test showed that *Lacticaseibacillus casei* CGMCC1.8727 had good bacteriostasis against *Vibrio parahaemolyticus*, which laid a foundation for the subsequent animal experiments.

SD rats aged 7 weeks were selected for the animal tests. Because the microcapsules of *Lacticaseibacillus casei* CGMCC1.8727 were too large for small experimental animals, it was difficult to deliver the tested components by microcapsule but was relatively easy to administer them by gavage. In the formal experiment, we used a number 11 gavage to administer gavage to the experimental animals. In the pre–experiment, the challenge concentration of *Vibrio parahaemolyticus* RIMD 2,210,663 strain, the administration concentration of probiotics, and the minimum safe treatment concentration of antibacterial peptides were determined. In the formal animal test, 5 g/l streptomycin was administered to the animals for 24 h, 2 days in advance of the test to clear the intestinal microbiota and reduce its impact. Fasting 1 day in advance of testing was conducive to emptying the residual food in the intestine and improving the possibility of *Vibrio parahaemolyticus* RIMD 2,210,663 contacting the intestinal mucosa. Twenty minutes before the challenge, 1 mL of 5% NaHCO_3_ was administered to neutralize the gastric acid in rat stomachs and reduce its damage to *Vibrio parahaemolyticus* RIMD 2210663. The above three experimental steps effectively improved the success rate of the modeling. An ELISA kit was used to detect the levels of TNF–α, IL–6, IL–8, and IL–1 β in the supernatant of rat intestinal tissue. TNF–α is a multifunctional inflammatory cytokine that stimulates IL–1 β and IL–6 release [30]. IL–6, a cytokine with a wide range of biological effects, can be secreted by macrophages, fibroblasts, T lymphocytes, and other cells [31]. IL–6 controls many physiological processes under basic and neuroinflammatory conditions [32,33]. The therapeutic effects of microencapsulated *Lacticaseibacillus casei* CGMCC1.8727, free probiotics, and combined drugs were compared. The concentration of *Vibrio parahaemolyticus* RIMD2210663 in rat feces was used to indicate the therapeutic effect of each treatment. The duration of *Lacticaseibacillus casei* CGMCC1.8727 in the feces of the rats in the microencapsulated *Lacticaseibacillus casei* CGMCC1.8727 group was longer compared with that in the other groups, which was caused by the slow release of microcapsules. The higher the concentration of *Vibrio parahaemolyticus* RIMD 2,210,663 in rat feces, the slower the recovery and the worse the curative effect, whereas the faster the recovery, the better the curative effect. The results showed that the in vivo therapeutic effect of microencapsulated probiotics was better than that of the free probiotics, and microencapsulated probiotics in combination with AMPs showed the strongest therapeutic effect.

The ratio of villi height to recess depth (v/c) is an important index to measure intestinal digestion and absorption function. The v/c ratio is commonly used to measure intestinal function. If the v/c ratio is reduced, the intestinal digestion and absorption capacity are reduced; if the v/c ratio is high, the intestinal mucosa is in good condition and the intestinal digestion and absorption function are strong. The results from duodenal pathological sections and the v/c ratio showed that compared with other treatment groups, the combined application of microcapsules and AMPs could effectively restore the intestinal villi damage and inflammatory reaction caused by *Vibrio parahaemolyticus* colonisation.

Total bacterial DNA was extracted from rat fecal samples for PCR amplification, and the v3–v4 region of the 16S rDNA gene was sequenced by the Illumina HiSeq 2500 platform to obtain biological information about the intestinal microbiota. OTU analysis and structural diversity analysis were carried out via information mining of the literature think tank to analyze the richness, diversity, and distribution of the flora in rat feces in the experimental groups. The results showed that the 6 h group had significantly increased abundances and diversity of flora, increased beneficial bacteria, and reduced harmful bacteria, and the recovery effect of intestinal microbiota was better. In recent years, scientific research has confirmed that changes in the composition of the intestinal microbiota affect the health of the host and that abnormalities in the digestive tract flora are related to the occurrence of colorectal cancer [34] and metabolic diseases [35]. Previous studies have also revealed that the probability of intestinal microbiota disorder and gastrointestinal maladjustment symptoms increases with an increasing level of peripheral serum inflammatory factors in asthmatic children [36].

This study showed that microencapsulated *Lacticaseibacillus casei* CGMCC1.8727 combined with Bomidin can effectively treat intestinal inflammation caused by *Vibrio parahaemolyticus* infection, and has a certain recovery and reconstruction effect on intestinal microflora. However, the specific molecular mechanism of microencapsulated *Lacticaseibacillus casei* CGMCC1.8727 combined with Bomidin on intestinal microflora reconstruction remains unclear. Its effective components and whether there is the interaction between components remain to be studied. Since the experimental period of this study was short, it is necessary to test whether microencapsulated *Lacticaseibacillus casei* CGMCC1.8727 combined with Bomidin has a more long–term effect on the recovery of intestinal microbiota in rats. In addition, the operation of spaced medication is relatively complicated, so the two drugs can be effectively combined and successively released by using related technologies, which are more convenient to operate, and lay a foundation for the application in production practice.

## Figures and Tables

**Table 1:** Orthogonal test of microcapsule preparation of *Lacticaseibacillus casei* CGMCC1.8727.

NO.	A	B	C	D	E	F	G
1	0.60%	6.0	1:6	1:5	1:2	40 min	1.20%
2	0.60%	5.5	2:3	1:5	1:8	50 min	1.00%
3	0.30%	5.5	1:4	1:10	1:6	30 min	1.20%
4	1.20%	5.5	2:3	1:8	1:8	60 min	0.60%
5	0.90%	5.5	1:8	1:20	1:2	60 min	0.80%
6	0.30%	6.0	1:4	1:15	1:8	40 min	1.00%
7	0.90%	6.0	1:4	1:20	1:8	30 min	0.60%
8	0.60%	4.5	1:4	1:10	1:4	50 min	0.80%
9	1.20%	5.0	1:8	1:5	1:6	30 min	1.00%
10	0.90%	4.5	1:6	1:15	1:6	60 min	1.00%
11	0.30%	5.5	1:8	1:15	1:2	50 min	1.20%
12	0.30%	5.0	1:6	1:5	1:8	60 min	0.80%
13	1.20%	4.5	1:4	1:5	1:4	60 min	1.20%
14	0.60%	5.0	1:4	1:15	1:2	60 min	0.60%
15	0.90%	6.0	1:8	1:5	1:4	50 min	0.60%
16	0.30%	4.5	1:6	1:20	1:6	50 min	0.60%
17	0.90%	4.5	2:3	1:10	1:2	40 min	1.00%
18	1.20%	6.0	1:6	1:10	1:2	30 min	0.80%
19	0.30%	5.0	2:3	1:20	1:4	40 min	0.80%
20	0.90%	5.0	1:6	1:10	1:8	50 min	1.20%
21	1.20%	6.0	2:3	1:15	1:6	50 min	0.80%
22	0.60%	5.0	1:8	1:10	1:6	40 min	0.60%
23	0.90%	5.5	1:4	1:5	1:6	40 min	0.80%
24	1.20%	5.5	1:6	1:15	1:4	40 min	0.60%
25	1.20%	4.5	1:4	1:10	1:4	40 min	0.80%
26	0.90%	5.0	2:3	1:15	1:4	30 min	1.20%
27	0.60%	6.0	2:3	1:20	1:6	60 min	1.20%
28	0.60%	5.5	1:6	1:20	1:4	30 min	1.00%
29	0.30%	4.5	2:3	1:5	1:2	30 min	0.60%
30	1.20%	5.0	1:4	1:20	1:2	50 min	1.00%
31	0.30%	6.0	1:8	1:10	1:4	60 min	1.00%
32	0.60%	4.5	1:8	1:15	1:8	30 min	0.80%

A, chitosan pH; B, chitosan concentration; C, proportion of mixed glue; D, bacterial colloid volume ratio; E, volume ratio of mycelium to chitosan solution; F, stirring time; G, concentration of mixed glue.

**Table 2:** Identification of primers for *Vibrio parahaemolyticus*.

Name	Primer Sequence (5′–3′)
VP16srRNA–F	AGAGTTTGATCCTGGCTCAG
VP16srRNA–R	TACGGCTACCTTGTTACGACTT
*tdh*–F	GTAAAGGTCTCTGACTTTTGGAC
*tdh*–R	TGGAATAGAACCTTCATCTTCACC
*tlh*–R	AAAGCGGATTATGCAGAAGCACTG
*tlh*–F	GCTACTTTCTAGCATTTTCTCTGC

**Table 3:** Orthogonal test results.

No.	EE (%)	No.	EE (%)	No.	EE (%)	No.	EE (%)
1	58.83 ± 2.15	9	80.45 ± 0.76	17	81.90 ± 1.41	25 *	93.12 ± 1.44
2	60.72 ± 1.16	10	65.75 ± 1.29	18	67.76 ± 0.72	26	65.78 ± 1.36
3	41.05 ± 0.39	11	72.81 ± 0.17	19	53.45 ± 0.48	27	42.15 ± 1.96
4	61.98 ± 1.04	12	67.54 ± 1.07	20	92.04 ± 1.06	28	56.84 ± 0.95
5	45.08 ± 0.92	13	58.22 ± 1.34	21	66.36 ± 0.59	29	72.89 ± 0.18
6	68.89 ± 0.84	14	75.99 ± 1.56	22	77.11 ± 1.01	30	74.12 ± 1.40
7	52.66 ± 0.57	15	62.02 ± 0.91	23	51.68 ± 2.06	31	56.77 ± 1.18
8	68.90 ± 0.66	16	61.13 ± 0.68	24	78.33 ± 0.74	32	67.48 ± 0.78

Data of EE are shown in mean ± SD (*n* = 3). EE, encapsulation efficiency; *, preparation parameters of the highest encapsulation efficiency.

**Table 4:** Measurement results of the bacteriostatic zone.

	10^7^ CFU/mL	10^8^ CFU/mL	10^9^ CFU/mL
Size of inhibition zone (mm)	1.58 ± 0.23	15.16 ± 0.32	20.12 ± 0.22
sensitivity	low–alcohol sensitivity	medium sensitivity	highly sensitivity

**Table 5:** Changes of villus height/crypt depth in duodenal and intestinal of rats after treatment.

Day	Groups					
	YL	WN	KJT	6 h	9 h	Negative
1	3.22 ± 0.13	3.90 ± 0.03	3.52 ± 0.08	3.17 ± 0.06	3.08 ± 0.06	4.74 ± 0.13
9	4.14 ± 0.09	4.89 ± 0.30	4.39 ± 0.11	5.05 ± 0.08	4.82 ± 0.09	4.93 ± 0.11

Villus height/crypt depth results were expressed as mean ± SD (*n* = 3). YL group: naked bacteria group; WN group: microcapsule group; KJT group: antibacterial pep–tide group; 6 h group: antibacterial peptide + microcapsule interval 6 h group; 9 h group: antibacterial peptide + microcapsule interval 9 h group; negative group: the control group.

**Table 6:** Intestinal supernatant inflammatory factor detection data.

Day	Groups						
	YL (pg/mL)	WN (pg/mL)	KJT (pg/mL)	6 h (pg/mL)	9 h (pg/mL)	Positive (pg/mL)	Negative (pg/mL)
A1	115.44 ± 4.61	157.68 ± 9.54	172.02 ± 4.01	120.51 ± 5.22	157.65 ± 7.97	181.85 ± 2.67	69.32 ± 3.5
A2	106 ± 4.31	114.64 ± 4.54	108.14 ± 2.69	96.27 ± 2	107.55 ± 2.48	167.51 ± 2.82	62.35 ± 4.25
A3	95.61 ± 4.49	92.07 ± 6.08	88.83 ± 2.38	83.69 ± 2.71	101.13 ± 1.62	157.38 ± 2.64	61.9 ± 4.15
A6	82.41 ± 2.58	82.97 ± 2.28	83.44 ± 4.15	76.14 ± 1.64	82.06 ± 1.68	169.1 ± 1.39	64.72 ± 2.46
A10	71.85 ± 1.52	69.66 ± 3.4	73.95 ± 3.1	69.54 ± 3.02	68.07 ± 2.76	109.6 ± 7.31	65.54 ± 1.65
B1	285.15 ± 9.68	263.31 ± 7.34	284.92 ± 9.99	252.93 ± 23.09	358.06 ± 9.6	217.03 ± 8.19	44.41 ± 5.72
B2	191.07 ± 6.04	158.29 ± 11.82	191.94 ± 6.13	109.04 ± 1.73	178.16 ± 10.6	362.14 ± 20.9	45.54 ± 4.49
B3	192.18 ± 4.8	110.17 ± 7.04	151.47 ± 3	91.96 ± 4.13	82.13 ± 1.52	284.62 ± 6.03	47.83 ± 3.97
B6	114.86 ± 9.48	90.54 ± 2.24	115.47 ± 5.5	78.34 ± 2.73	71.88 ± 1.74	247.95 ± 12.98	48.82 ± 1.19
B10	70.59 ± 6.47	46.73 ± 5.06	64.56 ± 5.2	54.26 ± 3.47	47.11 ± 3.77	116.05 ± 6.8	40.32 ± 2.89
C1	58.35 ± 2.28	66.91 ± 2.43	62.17 ± 5.14	47.63 ± 3.56	61.71 ± 2.73	42.61 ± 2.73	13.26 ± 1.01
C2	31.78 ± 2.48	27.29 ± 2.06	44.43 ± 0.99	30.29 ± 2.01	31.62 ± 1.14	53.42 ± 9.14	11.29 ± 0.82
C3	27.47 ± 2.9	23.41 ± 1.28	21.79 ± 4.5	17.02 ± 2.03	14.6 ± 2.48	50.68 ± 5.14	10.22 ± 0.82
C6	33.33 ± 0.8	11.48 ± 1.03	16.38 ± 3.7	13.25 ± 1.81	12.22 ± 1.22	31.06 ± 7.1	12.64 ± 0.92
C10	18.11 ± 0.68	11.17 ± 0.07	15.77 ± 0.5	11.94 ± 1.55	10.63 ± 1.37	31.98 ± 10.01	13.89 ± 1.43
D1	160.28 ± 14.3	151.04 ± 6.31	187.16 ± 8.53	130.54 ± 5.65	146.56 ± 6.14	199.45 ± 4.45	41.36 ± 0.86
D2	93.68 ± 4.26	75.36 ± 2.86	152.84 ± 3.58	89.21 ± 9.06	133.54 ± 3.7	227.38 ± 61.01	38.03 ± 1.9
D3	73.74 ± 4.33	60.36 ± 2.10	68.01 ± 2.16	62.06 ± 2.98	71.4 ± 2.24	115.89 ± 7.73	36.86 ± 1.14
D6	62.4 ± 1.14	47.66 ± 1.17	66.13 ± 1.57	53.49 ± 2.73	66.45 ± 2.35	84.35 ± 5.12	39.08 ± 3.02
D10	53.13 ± 3.09	34.10 ± 0.96	52.87 ± 2.6	40.65 ± 4.3	39.63 ± 2.59	82.77 ± 2	37.12 ± 4.05

Data are shown in mean ± SD (*n* = 3). YL group: naked bacteria group; WN group: microcapsule group; KJT group: antibacterial peptide group; 6 h group: antibacterial peptide + microcapsule interval 6 h group; 9 h group: antibacterial peptide + microcapsule interval 9 h group; positive group: rats in a normal saline group; negative group: the control group.

**Table 7:** *Vibrio parahaemolyticus* RIMD 2,210,663 concentration change data.

Day	Groups					
	YL lg (CFU/g)	WN lg (CFU/g)	KJT lg (CFU/g)	6 h lg (CFU/g)	9 h lg (CFU/g)	Negative lg(CFU/g)
1	7.47 ± 0.28	6.9 ± 0.14	7.65 ± 0.11	7.34 ± 0.22	8.67 ± 0.51	0
2	6.85 ± 0.34	5.73 ± 0.24	7.31 ± 0.39	6.44 ± 0.26	7.45 ± 0.28	0
3	6.73 ± 0.29	5.69 ± 0.08	5.64 ± 0.31	2.42 ± 0.41	5.65 ± 0.32	0
4	5.36 ± 0.48	2.12 ± 0.18	1.34 ± 0.15	0	1.09 ± 0.03	0
5	2.05 ± 0.14	0	0	0	0	0
6	0	0	0	0	0	0

Data are shown in mean ± SD (*n* = 3). YL: naked bacteria group; WN: microcapsule group; KJT: antibacterial peptide group; 6 h: antibacterial peptide + microcapsule interval 6 h group; 9 h: antibacterial peptide + microcapsule interval 9 h group; negative: the control group.

**Table 8:** Effective sequence data statistics of each sample.

Sample	Barcode	SeqNum	BaseNum	MeanLen	MinLen	MaxLen
WN–1	TTCCAT	89669	37107766	413.83	361	467
YL–1	TCCTGT	49005	20653859	421.46	358	463
KJT–1	GAAGGC	45220	18915799	418.31	388	465
6 h–1	ATGTCA	58962	24649490	418.06	354	461
9 h–1	CGGTTA	70867	29535906	416.78	362	465
WN–9	CTATAC	91653	38183432	416.61	358	443
YL–9	AGGAAC	60873	25518039	419.2	357	470
KJT–9	TTGCTC	45663	18968502	415.4	357	472
6 h–9	TGCTTA	40792	17076309	418.62	356	446
9 h–9	AGTGGC	44446	18704699	420.84	368	467
negative	TATTCT	64246	27004824	420.33	373	460
positive	AGAGTA	72845	30729200	421.84	362	476

YL group: naked bacteria group; WN group: microcapsule group; KJT group: antibacterial peptide group; 6 h group: antibacterial peptide + microcapsule interval 6 h group; 9 h group: antibacterial peptide + microcapsule interval 9 h group; positive group: rats in a normal saline group; negative group: the control group.

**Table 9:** Alpha diversity analysis of fecal flora.

Sample	Shannon	Chao	ACE	Simpson	Shannoneven	Coverage
6 h–1	2.622829	220.05	213.0943	0.163929	0.503472	0.999063
6 h–9	2.954129	256	262.5695	0.114476	0.546335	0.998562
9 h–1	1.923763	207.3704	215.237	0.277893	0.371659	0.999251
9 h–9	2.176321	201	211.22	0.190497	0.417761	0.998963
KJT–1	2.255237	238.6667	221.9981	0.201234	0.436175	0.998695
KJT–9	2.95379	269.25	261.0984	0.118612	0.54019	0.998802
WN–1	1.939884	236.4286	236.5196	0.383198	0.359966	0.999557
WN–9	3.75668	264.0526	265.6699	0.048502	0.677468	0.999689
YL–1	3.018849	239.2759	244.2592	0.081175	0.564075	0.998813
YL–9	2.922499	256.6207	255.9447	0.112485	0.527034	0.998911
negative	2.591384	255.4545	245.243	0.145064	0.488179	0.998855
positive	2.745618	182.7143	182.6732	0.104154	0.537094	0.999385

YL group: naked bacteria group; WN group: microcapsule group; KJT group: antibacterial peptide group; 6 h group: antibacterial peptide + microcapsule interval 6 h group; 9 h group: antibacterial peptide + microcapsule interval 9 h group; positive group: rats in a normal saline group; negative group: the control group.

## Data Availability

Not applicable.
